# Prediction of the Interface Behavior of a Steel/CFRP Hybrid Part Manufactured by Stamping

**DOI:** 10.3390/ma17174291

**Published:** 2024-08-30

**Authors:** Jae-Chang Ryu, Chan-Joo Lee, Do-Hoon Shin, Dae-Cheol Ko

**Affiliations:** 1 Department of Nanomechatronics Engineering, Pusan National University, Busan 46241, Republic of Korea; 2 Precision Manufacturing & Control R&D Group, Korea Institute of Industrial Technology, Jinju 52845, Republic of Korea; 3 Aerospace Engineering Team, Koreanair R&D Center, Busan 46712, Republic of Korea

**Keywords:** hybrid part, spring-back, stamping process, cohesive properties

## Abstract

Carbon fiber-reinforced plastic (CFRP) is a lightweight material. The automotive industry has focused on producing a steel/CFRP hybrid part to reduce overall weight. After manufacturing, delamination can occur at the interface between the CFRP and steel owing to the hybrid part constituting dissimilar materials. However, most studies have focused only on designing the manufacturing processes for the hybrid part or evaluating the adhesive used at the interface. Therefore, it is necessary to predict the behavior of the interface after demolding the hybrid part. This study aimed to predict the interface behavior of a steel/CFRP hybrid part by considering its forming and cohesive properties. First, double cantilever beam (DCB) and end-notched flexure (ENF) tests were performed to obtain cohesive parameters, such as energy release rate of modes I and II (G_I_, G_II_). The experimentally obtained properties were applied to the bonding area of the hybrid part. Subsequently, a forming simulation was performed to obtain the stress of the steel blank in the hybrid part. The stress distribution after forming was utilized as the initial condition for spring-back simulation. Finally, the interface behavior of the hybrid part was predicted by a spring-back simulation. The simulation was conducted using the residual stress of steel outer and the cohesive properties on the interface, without the application of any external forces. The cases of spring-back simulation were divided as delamination occurrence and attached state. The simulation results for prediction of delamination occurrence and bonding showed good agreement in both cases with experimental ones. The proposed method would contribute to expanding the manufacturing of the hybrid part by stamping and reducing the manufacturing cost by prediction of delamination occurrence.

## 1. Introduction

Recently, there has been an increased effort to study different ways to improve fuel efficiency and overcome environmental pollution in the automotive industry. One of the effective solutions to improve fuel efficiency is making the vehicle lightweight. Carbon fiber-reinforced plastic (CFRP) is a lightweight material with higher specific strength and stiffness than steel materials. In addition, CFRP is typically stronger than other FRP materials, such as basalt fiber-reinforced plastic (BFRP) and glass fiber-reinforced plastic (GFRP) in terms of strength and stiffness [1]. Therefore, many studies have been conducted on the application of CFRP materials in automotive parts [2,3,4]. Bastovansky et al. investigated flexural properties in different multi-layer CFRP [5]. They compared the experiments for four types of twill weave CFRP with a MF (mean field) model and a FE (finite element) model, and confirmed that the MF model was more accurate.

Steel/CFRP hybrid parts have been investigated in various studies because of their several advantages, including high strength and lightweight. Qihua et al. investigated the transverse crashworthiness characteristics of a steel/CFRP tube manufactured using filament winding [6]. They discussed the effects of the winding sequence, number of layers, and bending collapse behavior on the plastic deformation of the hybrid part using bending tests. Consequently, a modified theoretical model for bending prediction was proposed for the elastic and plastic regions. However, the proposed model was limited to hybrid tubes. Kim et al. designed a hybrid B-pillar to improve crashworthiness through design optimization and impact analysis [7]. In addition, they produced a hybrid part by assembling a composite reinforcement and steel outer with an adhesive. Drop-tower tests of the conventional and hybrid parts were conducted, and it was confirmed that the crashworthiness of the hybrid B-pillar improved, as expected in the simulation. However, the manufacturing process employed was not economical because of the necessity of the assemble process for the manufactured parts. Taylor et al. manufactured a hybrid part using a one-step process, which was compared with a traditional manufacturing process [8]. Hybrid parts fabricated through a one-step process with economic advantages indicated slightly lower flexural stiffness and strength but higher strain to failure and higher energy absorption. Kim et al. designed manufacturing processes using finite element (FE) simulations and manufactured a steel/CFRP hybrid part using prepreg compression molding (PCM) [9]. The hybrid part was produced by forming a CFRP reinforcement on a hot-stamped B-pillar to eliminate the additional assembly process. However, their manufacturing process comprised two stages because it involved forming a steel outer and forming CFRP on the preformed steel outer. Lee et al. manufactured a steel/CFRP hybrid part without preformed components [10]. A hybrid blank composed of a CFRP prepreg and steel sheet was employed to reduce the number of manufacturing processes. An FE simulation was conducted to investigate the contact pressure during the manufacturing process. The mechanical properties of the part deteriorated in the regions with low contact pressures. However, they did not discuss potential problems such as delamination of the interface between the CFRP and steel.

In the manufacturing of steel/CFRP hybrid part, delamination at the interface can occur because of the different material behaviors. During the manufacturing of the CFRP part, spring-in, which is the opposite deformation to the spring-back of the steel part, occurs when the formed part is demolded from the molds [11,12]. Many studies investigated strengthened hybrid parts consisting of dissimilar materials to predict the interface behavior [13]. Calabrese et al. investigated the pultruded CFRP–steel joint [14]. They employed a trapezoidal cohesive material law to model the bond behavior at interface and verified using a single-lap direct shear tests. Yuyang et al. introduced a bond-slip model of the steel/CFRP interface and investigated the effects of the adhesive type, adhesive thickness, and CFRP type on the interface behavior using the 3D-DIC method [15]. They reported that the adhesive thickness and inter-laminar shear properties of the CFRP plate significantly affected the bond-slip model, and verified the results through a comparison with experimental results. Abderrahim et al. investigated the behavior of a steel tube strengthened by a composite using the cohesive zone model (CZM), a well-known finite element model (FEM) to predict adhesive behavior [16]. They presented numerical and experimental results for 15 specimens with different composite thicknesses, and steel tube thicknesses and lengths. Zhen et al. developed a CZM with a user subroutine (UMAT) for the hybrid part [17]. A simulation of the single-lap test using the proposed method was performed, and the results were compared with the experimental results. Minnicino et al. performed an FE simulation of a microdroplet test to consider the progressive damage of the interphase using surface-based cohesive behavior, where the contact behavior theory is identical to the cohesive element used in the ABAQUS software [18]. In addition, they investigated the influence of various geometric parameters, such as blade opening, fiber-free length, and fiber diameter, on cohesive damage. However, most of these studies did not consider forming process of the hybrid part and focused on simple geometries. Hwang et al. investigated the effects of surface treatment on the bonding strength and spring-back of a CR980/CFRP hybrid part [19]. The increased bonding strength was dependent on the degree of surface treatment. Moreover, the spring-back angle of the hybrid part increased with the surface roughness. However, their study did not consider the interface behavior in the hybrid part. Park et al. predicted the interface behavior of a steel/CFRP hybrid part manufactured by stamping [20]. Double cantilever beam (DCB) and end-notched flexure (ENF) tests were conducted to obtain the energy release rate of the mixed mode, which was applied to the FE simulation using the CZM. However, they employed a displacement equal to the amount of spring-back experimentally measured as a boundary condition. In other words, their method was not suitable for applications involving complicated geometries.

This study aims to predict the interface behavior of a steel/CFRP hybrid part manufactured by stamping. First, DCB and ENF tests were performed to obtain the cohesive properties, such as the energy release rate of modes I and II. The properties obtained from the DCB and ENF tests were applied to the interface between the CFRP and steel. Second, a forming simulation of the steel/CFRP hybrid part was performed to obtain the residual stress in the steel part. The stress distribution of the steel obtained from forming simulation was used as a boundary condition to predict the delamination at the interface of the hybrid part during the spring-back simulation. Finally, a hat-shaped hybrid part was manufactured through stamping and compared with the FE results for verification.

## 2. Materials and Methods

### 2.1. Stamping Process for Manufacturing the Steel/CFRP Hybrid Part

**Figure 1:**
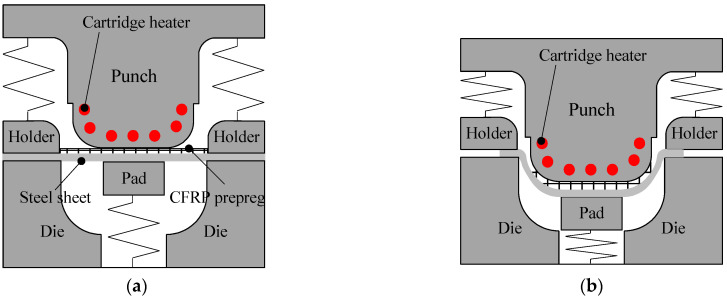

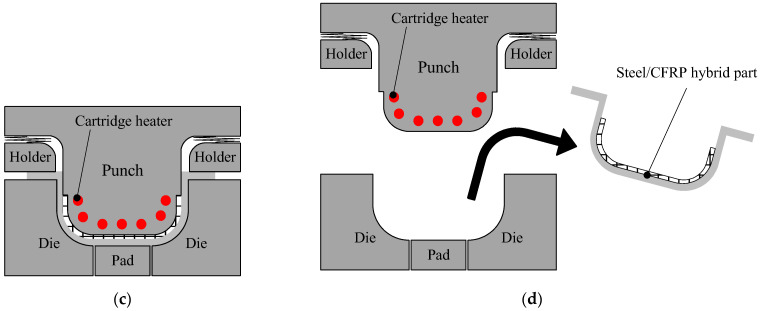
Manufacturing process of the steel/CFRP hybrid part: (**a**) positioning of hybrid blank on tool set; (**b**) forming; (**c**) curing and (**d**) demolding [20].

In this study, a stamping process was employed to manufacture steel/CFRP hybrid part. The CFRP reinforcement and steel outer were formed and assembled in a single step to reduce the number of manufacturing processes by the remove of additional assemble process [21]. Figure 1 shows the entire manufacturing process of the steel/CFRP hybrid part: (a) transferring the blank consisting of the CFRP patch and steel sheet to the lower die; (b) descending the punch preheated using cartridge heater to deform the blank, which is held by a pad and holders; (c) curing the formed part in the closed tool set; (d) demolding the hybrid part from the tool set, consisting of a punch, blank holder, lower die, and pad.

**Figure 2:**
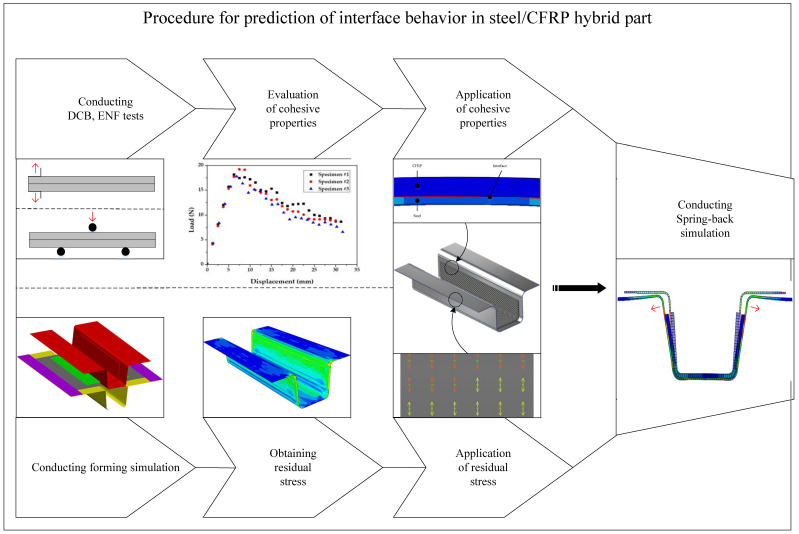
Procedure of FE simulation for prediction of the interface behavior of the hybrid part.

Generally, the deformation during the ejection of the formed composite part is spring-in, while that at the ejection of the formed steel part is spring-back, which occurs in the opposite direction. Therefore, delamination can occur at the interface of the hybrid part after manufacturing. However, many of the studies mentioned in Section 1 focused only on the forming of a hybrid part or the properties of the adhesive. They did not consider potential problems after manufacturing, such as delamination in the bonding area. Figure 2 illustrates the procedure of the FE simulation employed in this study to predict the delamination of the steel/CFRP hybrid part. First, the DCB and ENF tests were conducted to obtain the cohesive properties in the normal and shear directions. Second, a forming simulation was performed considering manufacturing conditions, to obtain the stress distribution of the steel material after stamping. Finally, the interface behavior of the hybrid part was predicted using a spring-back simulation. Here, the stress obtained from forming simulation was used as the initial condition. Additionally, the cohesive properties obtained from the DCB and ENF tests were applied to the bonding area of the hybrid part.

### 2.2. Evaluation of the Cohesive Properties at the Bonding Area

In general, the delamination in the bonding area occurred in the normal (mode I) and shear directions (mode II). In this study, DCB and ENF tests were performed to obtain the cohesive properties of the bonding area. The specimens were fabricated using a DP590 sheet (Hyundai Steel, Dangjin, South Korea) and CFRP (SK Chemicals, Seongnam, South Korea). The CFRP was a twill-weave prepreg and stocked in the 0° fiber direction. The specimens were cured for 3 min at 160 °C under the curing condition of the fast-cured epoxy. The release film was inserted between the steel plate and CFRP to create the initial crack for each test.

**Figure 3:**
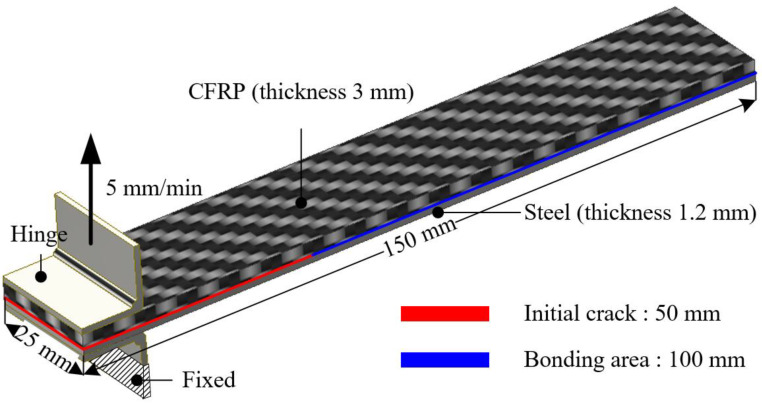
Dimension of specimen for DCB test.

A DCB test was performed to evaluate the cohesive properties of mode I. The specimens were fabricated according to ASTM 5528-13 [18]. Tests were conducted with 4 specimens using an INSTRON universal testing machine (INSTRON 5566A; Instron, Norwood, MA, USA). The velocity of the upper jig was 5 mm/min, while the lower jig held the lower hinge to restrict the vertical movement, as shown in Figure 3. 

The energy release rate of mode I (G_I_) is calculated by the modified beam theory (MBT), which is given by

(1) $$G_{I}=\frac{3P_{t}\delta_{t}}{2B(a_{0}+\left|\Delta \right|)}$$

where P_t_, δ_t_, B, and a_0_ are the maximum load, the displacement of the upper jig corresponding to the load, the specimen width, and the length of the separation, respectively. Δ is calculated by plotting the least squares of the cube root of compliance (C^1/3^) with respect to the delamination length [22].

**Figure 4:**
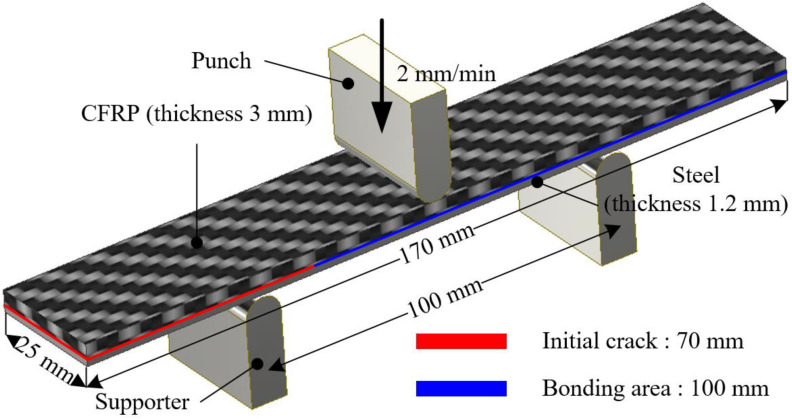
Dimension of specimen for ENF test.

ENF tests were conducted with 4 specimens to evaluate the cohesive properties of mode II. The specimens were fabricated for the experiments according to the ASTM D7905 standard [23]. The punch, at a velocity of 2 mm/min, descents to the specimen supported by the supporters, as shown in Figure 4. The energy release rate of mode II (G_II_) is expressed as

(2) $$G_{\mathrm{II}}=\frac{9a_{0}^{2}P_{c}\delta_{c}}{2B(2L^{3}+3a_{0}^{3})}$$

where a_0_, P_c_, δ_c_, B, and L are the crack length, load corresponding crack occurrence, displacement of punch at the crack occurrence, width of specimen, and distance between both supporters.

## 3. Prediction of the Interface Behavior of the Steel/CFRP Hybrid Part by Stamping

### 3.1. Verification of Cohesive Properties by FE Simulation

The cohesive zone model (CZM) is widely used to predict delamination of adhesive area. This method describes the fracture mechanics based on the traction-separation criterion. The damage initiation criterion is given by

(3) $${\left(\frac{<t_{n}>}{t_{n}^{0}}\right)}^{2}+{\left(\frac{t_{s}}{t_{s}^{0}}\right)}^{2}+{\left(\frac{t_{t}}{t_{t}^{0}}\right)}^{2}=1$$

Here, <> represents Macaulay brackets, indicating that a pure compression mode does not account for damage initiation. $t_{n},t_{s}$, and $t_{t}$ are the normal traction stress, first and second stress of shear direction, respectively. $t_{n}^{0},t_{s}^{0}$, and $t_{t}^{0}$ are the strength for the normal direction (mode I), first and second shear direction (mode II, III). Damage initiation occurs when the quadratic interaction function reaches 1. If the value of normal stress is negative ($t_{n}<0$), only the shear traction is considered in damage initiation. Once the damage initiation criterion reaches 1, the damage behavior is controlled by the damage evolution law until failure. Beyond the damage initiation, the damage evolution is described with damage variable D. The damage evolution model is as follows

(4) $$t_{n}=\left\{\begin{array}{r} \left(1-D\right)\bar{t_{n}},\;\bar{t_{n}}\geq 0\\ \bar{t_{n}},\;\bar{t_{n}}<0 \end{array}\right.\;t_{s}=\left(1-D\right)\bar{t_{s}}\;t_{t}=\left(1-D\right)\bar{t_{t}}$$

Here, $\bar{t_{n}},\bar{t_{s}}$ and $\bar{t_{t}}$ are the stress components predicted by the initial traction-separation behavior for the current strain before any damage occurs. To account for crack closure under compressive loads, the compressive stress does not contribute to damage for $\bar{t_{n}}<0$. The damage variable, D, progresses from 0 to 1 due to external loading after the damage initiation. When D reaches 1, the tractions in both normal and shear modes become zero.

**Figure 5:**
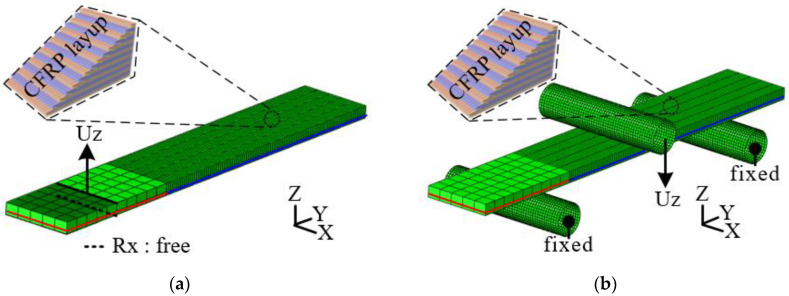
The FE model and boundary conditions of experiments: (**a**) DCB test; (**b**) ENF test.

In this study, FE simulations of the DCB and ENF tests were performed using the commercial FE software ABAQUS 2020 to verify the obtained cohesive properties. In the FE simulation, the energy release rate along the direction is an essential input parameter. Table 1 lists the mechanical properties of the CFRP and the energy release rates of modes I and II. The FE model and boundary conditions for each test are shown in Figure 5. The steel and CFRP were modeled using a 4-node rectangular shell mesh. In addition, the CFRP layup and steel thickness were applied to the element. In the DCB test, vertical displacement (U_Z_) was applied to the upper hinge. Fixed conditions except for the axial rotation (R_X_) of the hinge were applied to describe the rotational freedom of the hinge in the experiment, as shown in Figure 5a. The vertical displacement (U_Z_) of the punch and the fixed conditions of the supporters were employed in the FE model, similar to the actual ENF test, as illustrated in Figure 5b.

**Figure 6:**
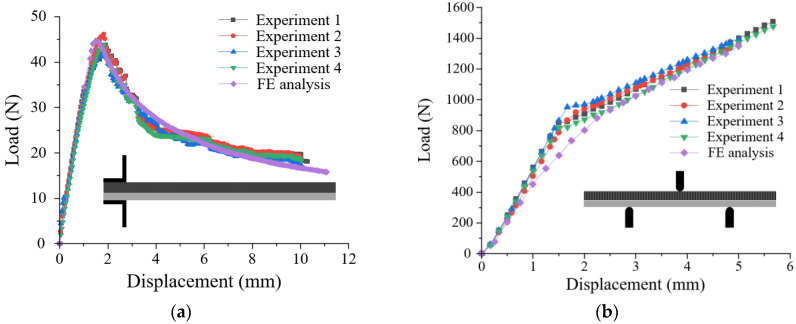
Comparison of FE simulation and experiments: (**a**) DCB test; (**b**) ENF test.

Figure 6 presents the results of the FE simulation compared with the experimental results of the DCB and ENF tests. The FE simulation using the cohesive properties of modes I and II was in good agreement with the experimental results in the load-displacement curve. Here, the displacement of DCB and ENF tests meant vertical displacement of the upper hinge and punch, respectively. The cohesive properties, verified by a comparison of the FE simulation and experiments were employed to predict the interface behavior of the hybrid part in Section 3.

### 3.2. Forming Simulation of the Steel/CFRP Hybrid Part

**Figure 7:**
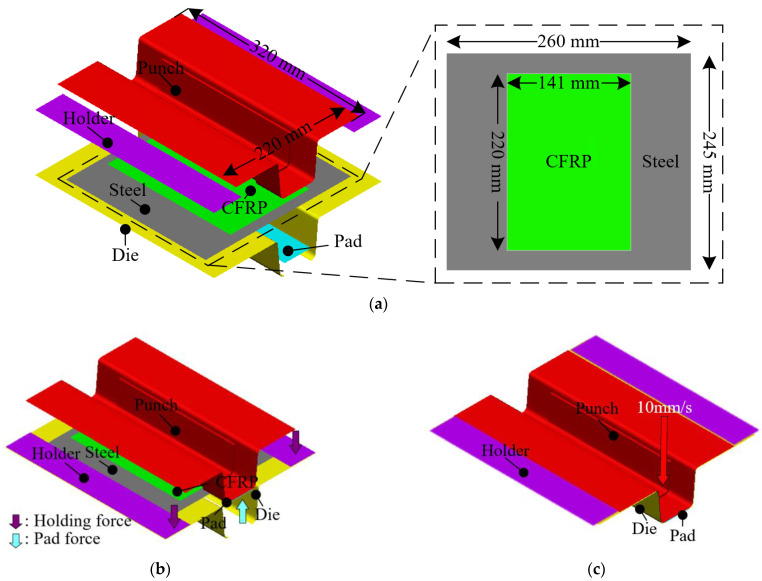
The FE model of the stamping process for manufacturing the steel/CFRP hybrid part: (**a**) initial state; (**b**) holding; (**c**) forming.

In this study, an FE simulation of the stamping process was performed considering the manufacturing conditions using the commercial FE software PAM-FORM 2020. Figure 7 shows the FE model of the stamping process, which was divided into the initial state, holding, and forming. In the initial state, a hybrid blank composed of a thermoset CFRP prepreg and a steel sheet was placed on the tool set, as shown in Figure 7a. Two types of steel sheets, DP590 and DP780, were utilized to investigate the effect of residual stress on delamination. DP590 and DP780 are same steel sheets and have almost identical mechanical properties, except for yield strength and tensile strength. Table 2 summarizes the mechanical properties of DP590 and DP780. Reference data to consider forming of prepregs were applied to the shear deformation properties of the CFRP [17]. In the FE model, the CFRP and steel were modeled using a 4-node rectangular shell element. The mesh sizes of the CFRP and steel were 2 and 4 mm, respectively. The thicknesses of the CFRP and steel were 3 and 1.2 mm, respectively. Holder and pad force of 11 kN and 13 kN were applied to hold the blank, as shown in Figure 7b. The velocity of the punch was 10 mm/s, and the die was fixed, as shown in Figure 7c. The stress distribution in the steel material during stamping was obtained from the simulation and then used as the initial condition in the spring-back simulation.

### 3.3. Spring-Back Simulation of the Hybrid Part

As mentioned in Section 2.1, deformations after stamping the CFRP and steel parts occurred in opposite directions. However, most studies on hybrid part have focused only on forming or interface behavior. They did not consider other potential problems, such as delamination after stamping. Therefore, the prediction of the interface behavior after manufacturing is important.

**Figure 8:**
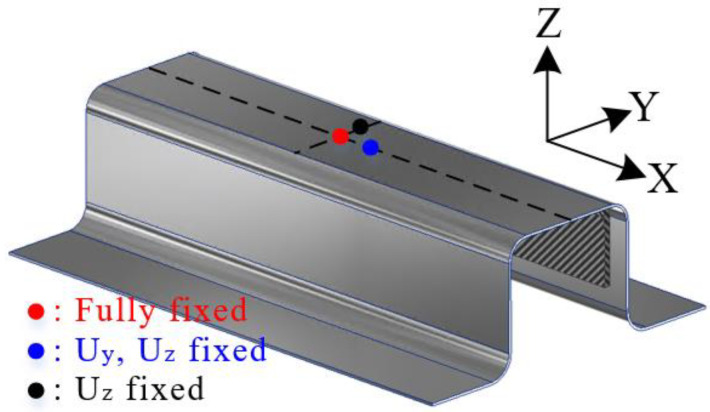
Boundary conditions for the spring-back simulation.

In this study, the interface behavior of the hybrid part was predicted using the results of forming and cohesive properties. First, the deformed shapes from the forming simulation were employed to predict delamination. The stress distribution of the steel part in PAM-FORM was transferred to ABAQUS using the user subroutine SIGINI, allowing users to define the initial stress in the model before the simulation. Cohesive properties were also applied at the interface of the hybrid part. Figure 8 shows the boundary conditions for the spring-back simulations. A fully fixed (translations and rotations in X, Y, and Z directions) constraint at the center point and two restricted points (translation in the Z direction for one, and translations in Y and Z directions for the other) were applied to prevent rigid body modes.

**Figure 9:**
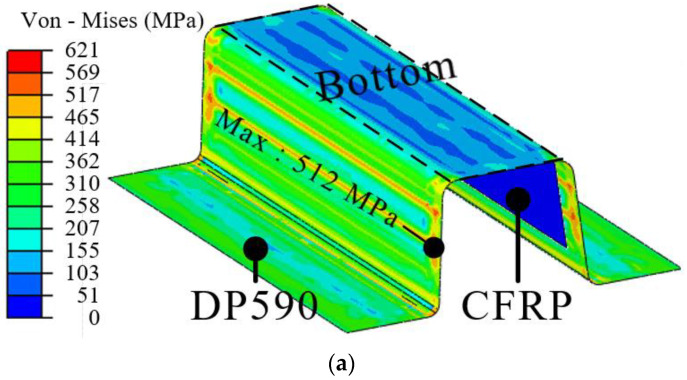

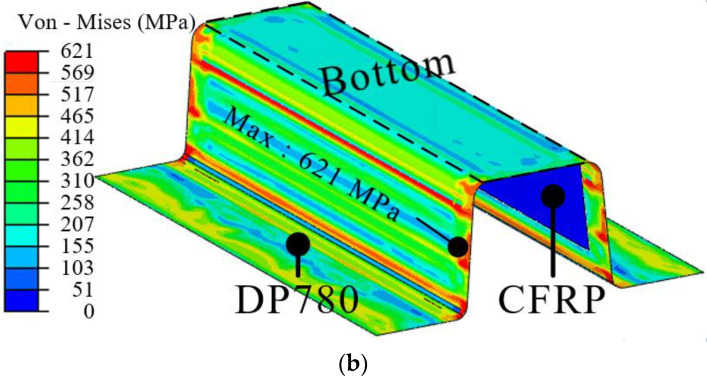
Stress distribution for spring-back simulation: (**a**) DP590; (**b**) DP780.

**Figure 10:**
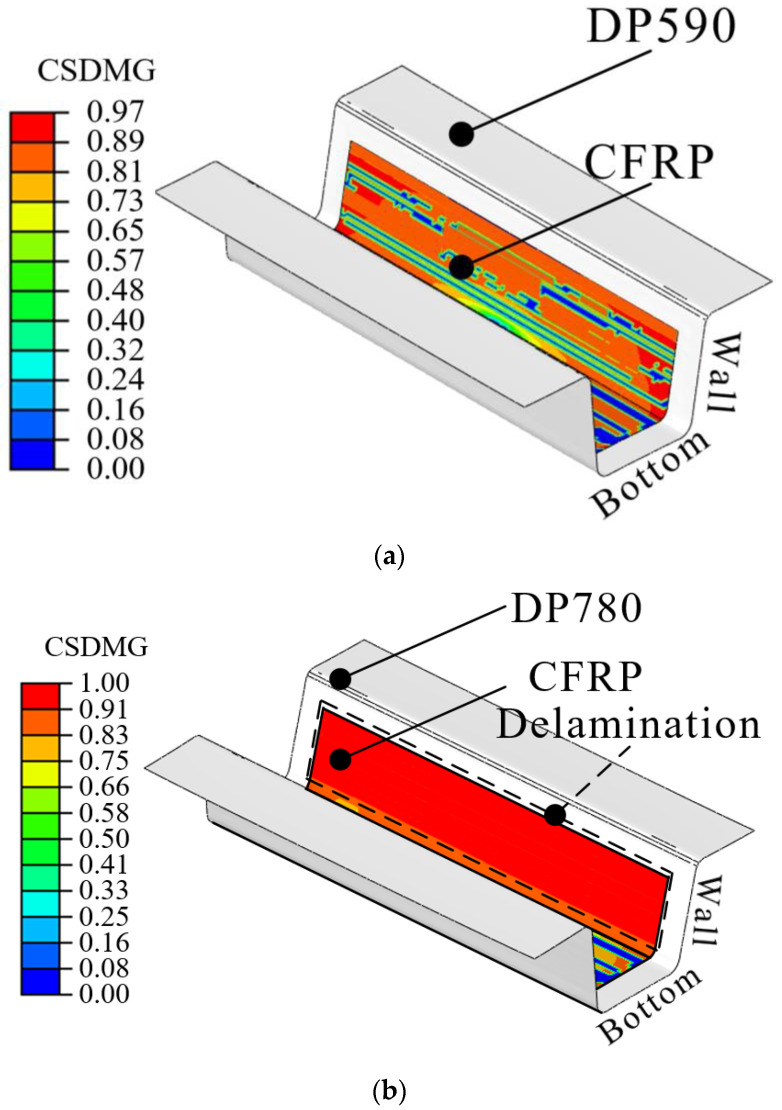
FE results of spring-back simulation: (**a**) DP590/CFRP; (**b**) DP780/CFRP.

**Figure 11:**
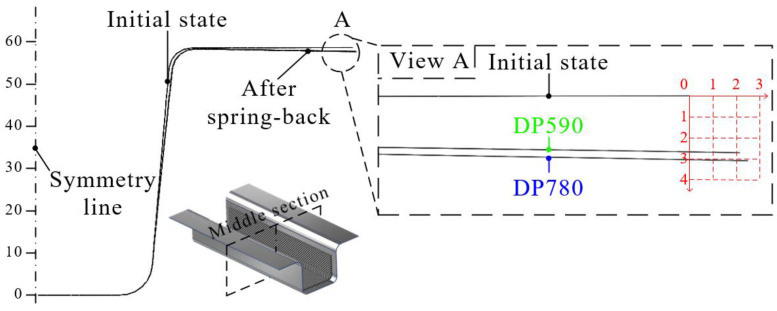
Comparison of the initial state and the spring-back state.

Figure 9 shows the applied stress distribution before performing the spring-back simulation. The stress distributions of DP590 and DP780 were used to perform spring-back simulations of DP590/CFRP and DP780/CFRP. The stress in the bottom area was lower than ones in other areas because the punch and pad held it from the beginning to the end of the forming process, resulting in minimal deformation. However, the stress at the wall was higher than ones at the bottom because various deformations, such as bending and tension, occurred during the forming process, as shown in Figure 7. Subsequently, a spring-back simulation was conducted to predict the interface behavior. Table 1 lists the cohesive properties applied to the bonding area. Although the experiments were performed using only DP590 steel, this was acceptable for DP780/CFRP because the DCB and ENF tests were performed within the elastic deformation region of the adherend. If the DCB and ENF tests were conducted using DP780/CFRP, the same cohesive properties cannot be applied to DP590/CFRP. Even if there is no plastic deformation of DP780, DP590 could be deformed under lower stress. In other words, if there is no plastic deformation in the adherend during experiments with using DP590/CFRP, the same properties are acceptable to DP780/CFRP. Additionally, surface treatments were not applied at interface between steel sheet and CFRP. Figure 10 presents the damage of interface after spring-back simulations. The damage value progresses from 0 to 1. When the value of damage reaches 1, it means the delamination occurs at the interface. The FE simulation predicted that delamination would not occur in the DP590/CFRP hybrid part because the maximum value of damage was 0.97, as shown in Figure 10a. However, for DP780/CFRP, debonding was predicted to occur at the wall of the hybrid part, as shown in Figure 10b. Compared to the wall, CFRP and steel were attached to the bottom of the hybrid part. Generally, the amount of spring-back increased with the increase in the strength of steel material. Figure 11 shows the shapes of the initial state and after spring-back in the middle section of the hybrid part. The steel outers moved outside, and the deformation value of DP780 was higher than that of DP590, as shown in view A.

## 4. Experimental Verifications

**Figure 12:**
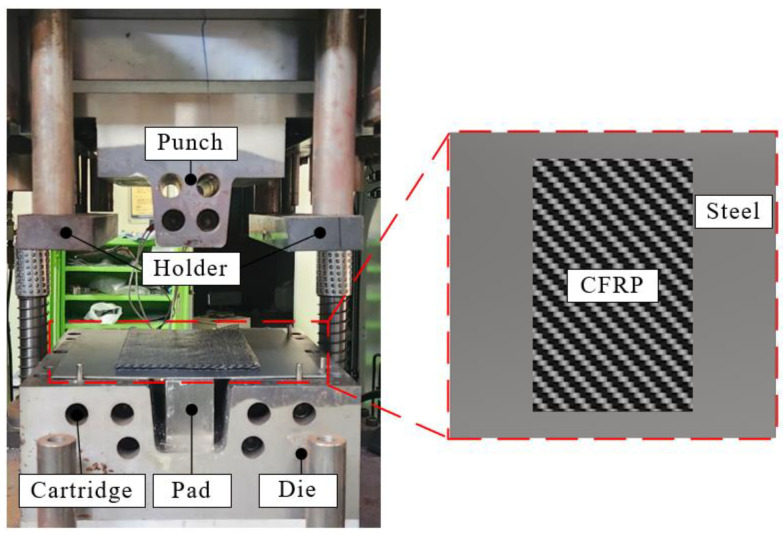
Experimental equipment to manufacture the steel/CFRP hybrid part.

In this study, steel/CFRP hybrid parts were manufactured to validate FE simulation results. The experimental cases were classified as DP590/CFRP and DP780/CFRP. Figure 12 illustrates the equipment used to produce hybrid parts through stamping. The tool set consisted of a punch with an inserted cartridge heater, blank holders, a lower die, and a pad placed on a 2000 kN servo press. The punch was preheated to 160 °C considering the curing condition of CFRP material. The hybrid blank was composed of steel and CFRP and placed on the die. Here, any surface treatment was not applied to interface of the hybrid part.

The manufacturing process of the steel/CFRP hybrid part is as follows. First, the liquid release agent was spread on the surfaces of the tool set to prevent CFRP from sticking to the molds. Subsequently, the preheated punch is moved down to form a blank held by the blank holders and pad. After forming, the hybrid part was cured within closed molds. Finally, the hybrid component was ejected after curing for 3 min.

**Figure 13:**
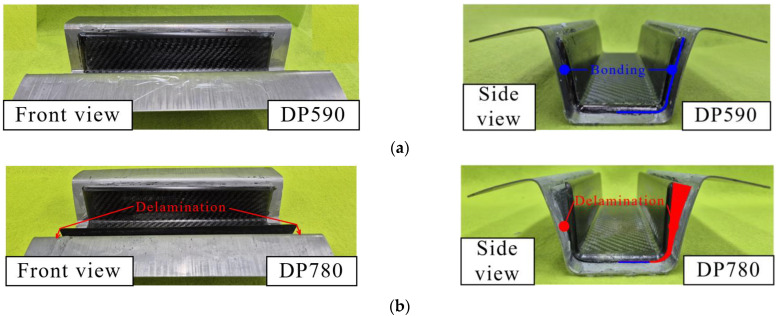
Hybrid part: (**a**) DP590/CFRP; (**b**) DP780/CFRP.

The experimental and FE simulation results were compared to verify the results predicted by the FE simulation. Figure 13 presents the experimental results obtained using DP590/CFRP and DP780/CFRP. In the DP590/CFRP hybrid part, steel and CFRP were attached to all the bonding areas, whereas delamination occurred at the interface in the DP780/CFRP hybrid part. The wall of the hybrid part was separated, as predicted by FE simulation in Figure 10b. However, some discrepancies were noted at the bottom surface, where partial delamination was observed experimentally, unlike the simulation results. Corner at the bottom, near the wall, was separated in the experiment, as shown in Figure 13b. In the simulation, the bottom remained bonded, whereas slight delamination occurred at the bottom in the experiment, as shown in Figure 10b and Figure 13b. The result of verification indicated that the additional consideration is required when using higher strength steels to prevent delamination. The prediction method of this study can be developed by investigating thermomechanical influences during curing and cooling on the interface behavior. The steel part is heated during curing and cooled after ejection. Therefore, the steel part shrink after being ejected from the toolset. However, the shrinkage of the thermoset composite is considerably small compared to that of steel. This indicates that the damage in the bonding area can progress due to the shrinkage of the outer steel. The accuracy of the spring-back simulation can be improved by investigating the thermal effects of delamination. These findings indicate the feasibility of the coupled analysis of forming simulation with cohesive properties to predict interface behavior, although improvements are needed for greater accuracy, particularly in the DP780/CFRP case. 

The proposed method can contribute to reduce the cost for producing hybrid part prior to actual manufacturing. If the manufacturing process is established, a coupled analysis can be utilized to predict the occurrence of delamination in the hybrid part. If the adhesive is predetermined, the analysis can be used to prevent delamination by optimizing the forming process parameters. This study enhances the reliability and quality of the parts by predicting delamination through the coupled analysis. This ensures better performance and lower defect rates in the final products.

In addition, this method can be used for ultralightweight hybrid parts consisting of other lightweight material such as aluminum. Generally, the amount of spring-back of aluminum part is greater than that of steel one due to the difference of mechanical properties. Therefore, the application of aluminum outer for the hybrid part is more challenging compared to steel outer. The proposed method can be used to predict the occurrence of delamination and optimize the forming process to accommodate the higher spring-back of aluminum, thereby facilitating the use of aluminum in ultralightweight hybrid part. Many researchers have evaluated the performance of lightweight product consists of aluminum [24,25]. However, evaluation of manufacture part should be carried out with consideration of the deformation occurred during ejecting. The proposed method in this study and structural analysis can make it possible to take advantage of the benefits of aluminum’s low weight while maintaining structural performance.

## 5. Conclusions

In this study, the interface behavior of a steel/CFRP hybrid part manufactured by stamping was predicted via FE simulation and verified through comparison with experimental results. The results of the FE simulation were as follows. First, the DCB and ENF tests were performed to evaluate the cohesive properties of modes I and II according to ASTM d5528 and d7905, respectively. The energy release rates of modes I and II obtained from the tests were 0.13 and 4.96 N/mm, respectively. Cohesive properties were applied to describe the interface behavior of the hybrid part. Second, a forming simulation was performed considering actual manufacturing conditions. A hybrid blank consisting of a CFRP patch and a steel sheet was employed for one-step forming. In the final step of the proposed method, a spring-back simulation was performed using the stress distribution of steel part and the obtained cohesive properties. Here, the stress distribution of the steel material at the end of forming was employed as the initial state from PAM-FORM to ABAQUS using the user subroutine SIGINI code. In addition, cohesive properties were applied to the bonding area to predict the interface behavior after ejecting the hybrid part. The FE simulation cases were classified as DP590/CFRP and DP780/CFRP. The FE simulation of DP590/CFRP predicted the state of bonded at the interface because the maximum value of damage was lower than 1. However, the FE results of the DP780/CFRP showed delamination at the sidewall of the hybrid part. Hybrid parts were manufactured using DP590/CFRP and DP780/CFRP to verify the FE approach. A comparison of both cases indicated that the proposed FE approach was reasonable and in good agreement. Therefore, the FE simulation performed in this study is appropriate to predict the interface behavior of the hybrid part manufactured via stamping.

## Figures and Tables

**Table 1:** Material properties of CFRP and bonding area used in FE simulation.

	Property	Value
CFRP	Elastic modulus in 0° direction	65.01 GPa
	Elastic modulus in 90° direction	65.01 GPa
	Shear modulus in 1–2 plane	12.69 GPa
	Shear modulus in 2–3 plane	1.38 GPa
	Shear modulus in 1–3 plane	1.38 GPa
	Poisson’s ratio	0.13
Bonding area	Energy release rate of mode I	0.13 N/mm
	Energy release rate of mode II	4.96 N/mm

**Table 2:** Mechanical properties of DP590 and DP780.

	DP590	DP780
**Elastic modulus**	210 GPa	
**Yield strength**	390 MPa	527 MPa
**Tensile strength**	679 MPa	957 MPa
**Blank thickness**	1.2 mm	

## Data Availability

The data presented in this study are available on request from the corresponding author and the first author on reasonable request.
